# Catalytic performance of Metal-Organic-Frameworks vs. extra-large pore zeolite UTL in condensation reactions

**DOI:** 10.3389/fchem.2013.00011

**Published:** 2013-08-28

**Authors:** Mariya Shamzhy, Maksym Opanasenko, Oleksiy Shvets, Jiří Čejka

**Affiliations:** ^1^J. Heyrovský Institute of Physical Chemistry, Department of Synthesis and Catalysis, Academy of Sciences of Czech RepublicPrague, Czech Republic; ^2^L.V. Pisarzhevskiy Institute of Physical Chemistry, Department of Porous Substances and Materials, National Academy of Sciences of UkraineKyiv, Ukraine

**Keywords:** condensation reactions, MOFs, zeolites, UTL, Prins reaction

## Abstract

Catalytic behavior of isomorphously substituted B-, Al-, Ga-, and Fe-containing extra-large pore UTL zeolites was investigated in Knoevenagel condensation involving aldehydes, Pechmann condensation of 1-naphthol with ethylacetoacetate, and Prins reaction of β-pinene with formaldehyde and compared with large-pore aluminosilicate zeolite beta and representative Metal-Organic-Frameworks Cu_3_(BTC)_2_ and Fe(BTC). The yield of the target product over the investigated catalysts in Knoevenagel condensation increases in the following sequence: (Al)beta < (Al)UTL < (Ga)UTL < (Fe)UTL < Fe(BTC) < (B)UTL < Cu_3_(BTC)_2_ being mainly related to the improving selectivity with decreasing strength of active sites of the individual catalysts. The catalytic performance of Fe(BTC), containing the highest concentration of Lewis acid sites of the appropriate strength is superior over large-pore zeolite (Al)beta and B-, Al-, Ga-, Fe-substituted extra-large pore zeolites UTL in Prins reaction of β-pinene with formaldehyde and Pechmann condensation of 1-naphthol with ethylacetoacetate.

## Introduction

Condensation reactions, in which a carbonyl group undergoes nucleophilic attack by the enol form or enolate carbanions, are powerful tools to form C-C bonds affording for easy preparation of useful organic compounds (Li, 2005). Among them, Knoevenagel, Pechmann, and Prins reactions, being quite sensitive to the nature of active sites of a catalyst, are particularly interesting to be studied over solid acids, containing active sites of different type and strength.

**Scheme 1:**
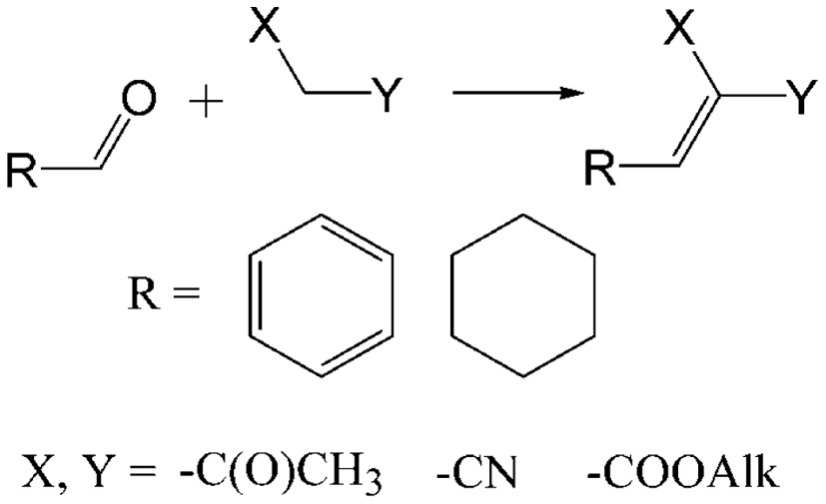
**Schematic representation of Knoevenagel condensation**.

Knoevenagel condensation of aldehydes with compounds containing active methylene group (Scheme 1) has a wide applications in the synthesis of fine chemicals (Freeman, 1981), biologically active substances (Lai et al., 2003), or precursors for hetero Diels–Alder reactions (Borah et al., 2005). Various homogeneous and heterogeneous catalysts were investigated in Knoevenagel condensation, namely TiCl_4_ (Green et al., 1985), ZnCl_2_ (Shanthan and Venkataratnam, 1991), MgF_2_ (Kumbhare and Sridhar, 2008), HClO_4_–SiO_2_ (Bartoli et al., 2006), Ni-SiO_2_ (Rajasekhar Pullabhotla et al., 2009), phosphates (Bennazha et al., 2003), zeolites (Corma et al., 1990; Corma and Martin-Aranda, 1993; Reddy and Verma, 1997; Joshi et al., 2003), and clays (Bigi et al., 1999). Heterogeneous catalysts provide number of advantages (they are easily recoverable, reusable and minimize the undesired wastes) over homogeneous ones but most examples of Knoevenagel condensation over solid catalysts are related to base-activated processes. Since that, comparative investigation of acid catalysts in Knoevenagel condensation seems topical.

**Scheme 2:**
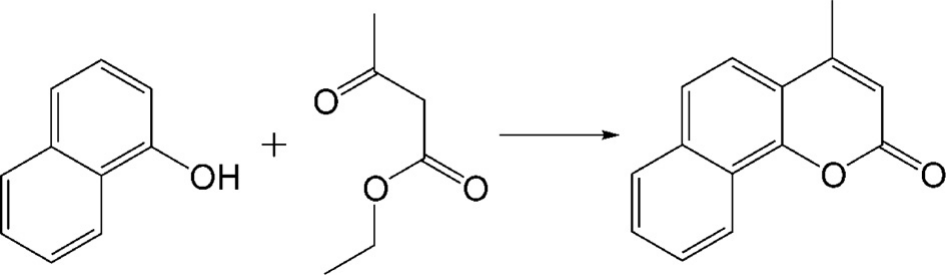
**Schematic representation of Pechmann condensation of 1-naphthol and ethyl acetoacetate**.

Pechmann condensation is a reaction of phenols with beta-ketonic esters or unsaturated carboxylic acids (Scheme 2) resulting in the formation of coumarins—important natural substances with broad applications in pharmaceutical, agrochemical, and fragrance industries (Weinmann, 1997). Pechmann reaction was carried out in the presence of concentrated H_2_SO_4_ (Russell and Frye, 1955), CF_3_COOH (Woods and Sapp, 1962), P_2_O_5_ (Canter et al., 1931), AlCl_3_ (Das Gupta et al., 1969). During the last decade, zeolites (Hoefnagel et al., 1995), amberlyst (Sabou et al., 2005), montmorillonite K 10 (Li et al., 1998), heteropolyacids (Torviso et al., 2008), functionalized mesoporous silica [e.g., Zr-TMS (Torviso et al., 2008), Al-MCM-41 (Sudha et al., 2008), SBA-15-Ph-Pr-SO_3_H (Karimi and Zareyee, 2008)], metal oxides (e.g., sulfated zirconia) (Tyagi et al., 2007), inorganic ion exchangers (Sabou et al., 2005), and superacid-functionalized mesoporous materials (Kalita et al., 2010) have also been employed to catalyze Pechmann condensation.

**Scheme 3:**
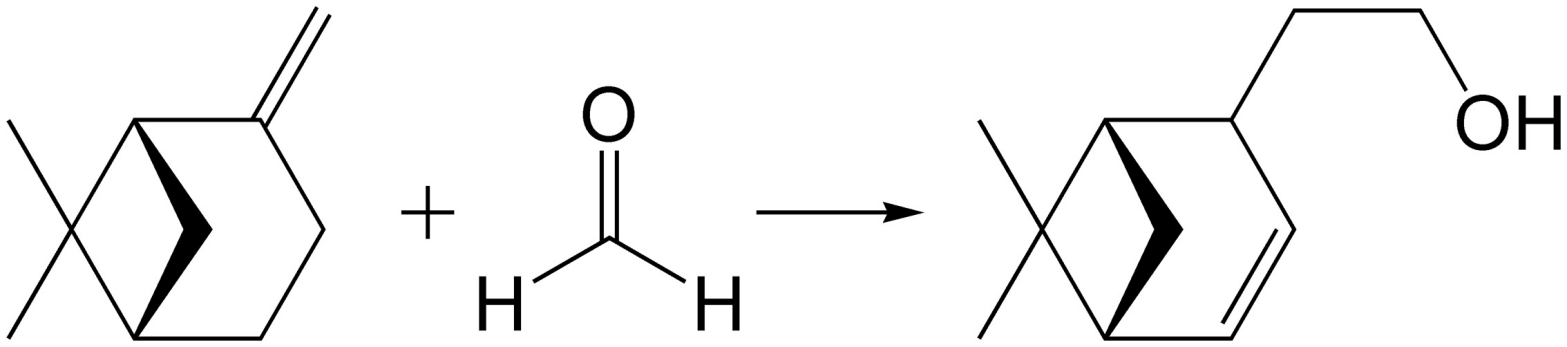
**Prins reaction of β-pinene with formaldehyde**.

Prins reaction, involving the electrophilic addition of an activated paraformaldehyde (PF) to β-pinene, leads to the formation of nopol (6,6-dimethylbicyclo[3.1.1]hept-2-ene-2-ethanol), an optically active bicyclic primary alcohol, useful in the agrochemical industry to produce pesticides, soap perfumes, detergents and polishes (Bledsoe, 1997) (Scheme 3). Hydrochloric acid, alkyl-substituted aluminum chlorides (Williams et al., 2002), SnCl_4_ (Andersen et al., 1985), InCl_3_ (Yadav et al., 2003), and heteropolyacids (Li et al., 2004) are typically used to catalyze Prins reaction in homogeneous systems. Several heterogeneous catalytic systems have also been reported for Prins condensation of β-pinene with formaldehyde, including mesoporous iron phosphate (Pillai and Sahle-Demessie, 2004), Fe–Zn double cyanide (Patil et al., 2007), metal supported (Zn-, Al- and Sn-) MCM-41 mesoporous molecular sieves (de Villa and Alarcon, 2002; Corma and Renz, 2007; Alarcon et al., 2010; Selvaraj and Sinha, 2010), SnCl_4_ grafted on MCM-41 (de Villa et al., 2005), ZnCl_2_ impregnated on Montmorillonite (Yadav and Jasra, 2006), and Sn-SBA-15 (Selvaraj and Choe, 2010). However, doping of metals into mesoporous silica does not produce single site catalysts and due to the amorphous nature, metal-doped mesoporous materials are not stable enough due to a leaching of the active phase.

Due to extra-high porosity (Chae et al., 2004), the regular arrangement of a large number of active sites and mostly Lewis acidity metal-organic frameworks (MOFs) attract significant attention as perspective heterogeneous catalysts, which are able to compete with the traditionally used zeolites (Li et al., 1999; Eddaoudi et al., 2002; Chae et al., 2004; Corma et al., 2010; Dhakshinamoorthy et al., 2013). MOFs exhibit a high catalytic activity in Knoevenagel condensation reactions (Opanasenko et al., 2013a) and perform even better in transformations of bulky substrates than zeolites due to steric limitations. At the same time, other condensation reactions have been considerably less explored over MOFs (Opanasenko et al., 2013b).

Extra-large pore zeolites, possessing micropores larger than 0.85 nm, represent another group of porous materials with a great potential for application in fine chemistry catalysis (Jiang et al., 2010). Zeolite UTL (Corma et al., 2004; Paillaud et al., 2004; Shvets et al., 2008), containing two-dimensional system of intersecting large (12-rings) and extra-large channels (14-rings) with dimensions 0.85 × 0.55 nm and 0.95 × 0.71 nm, belongs to the thermally stable extra-large pore zeolites. Moreover, the catalytic activity of (Al)UTL was shown to be higher with respect to UTD-1 zeolite, having one-dimensional 14-ring channel system, in dealkylation of tri-isopropylbenzene and di-isopropylbenzene (Corma et al., 2004). Recently, higher activity and selectivity of Al-substituted UTL zeolites in acylation of p-xylene with benzoyl chloride in comparison to large-pore alumosilicate zeolite beta was shown (Shamzhy et al., 2013). However, despite extra-large pore zeolite UTL looks like promising solid catalyst for the liquid phase condensation reactions (i.e., Knoevenagel, Pechmann), as far as we know, there are no reports addressing this issue.

Our contribution is aimed at the investigation of catalytic properties of some zeolites and metal-organic-frameworks, in particular in comparison of B-, Al-, Ga-, Fe-substituted extra-large-pore zeolites UTL with large-pore alumosilicate zeolite (Al)beta and MOFs [Cu_3_(BTC)_2_, Fe(BTC)] in Knoevenagel, Pechmann, and Prins reactions. The selection of (Al)beta, Cu_3_(BTC)_2_ and Fe(BTC) as the reference materials was made based on their wide availability and ample use as solid catalysts.

## Experimental section

### Materials and methods

1-naphthol (≥98.0%), cyclohexane carbaldehyde (97%), benzaldehyde (≥99.0%), ethyl acetoacetate (≥99.0%), β-pinene (99%) and PF (95%) were used as substrates, n-dodecane (≥99%) and mesitylene (≥99%)—as internal standards, nitrobenzene (99%), p-xylene (≥99%) and acetonitrile (99.8%)—as solvents in catalytic experiments. 2-ethylpiperidine (≥98.0%), 1,5-dibromopentane (≥98.0%), chloroform (≥99.0%), diethyl ether (≥99.0%), sodium sulfate anhydrous (≥99.0%) were used for the synthesis of structure directing agent. Boric acid (≥99.0%), aluminum hydroxide (reagent grade), iron(III) nitrate nonahydrate (≥98.0%), gallium(III) nitrate hydrate (99.9%), germanium(IV) oxide (99.9%), Cab-O-Sil M5 were used for the synthesis of UTL zeolites.

All reactants and solvents were obtained from Sigma Aldrich and used as received without any further treatment.

Ion-exchange resin AG 1-X8 was obtained from Bio-Rad.

### Synthesis of templates and catalysts

Preparation of 7-ethyl-6-azoniaspiro[5.5]undecane hydroxide was carried out using a method similar to Refs. (Shvets et al., 2010, 2011). The detailed description of the synthesis of UTL zeolites can be found in refs. (Shvets et al., 2010, 2011; Shamzhy et al., 2012). Zeolite (Al)beta was obtained from zeolyst in NH_4_-form and calcined at 450°C for 4 h prior to use.

Cu_3_(BTC)_2_ (Basolite C300) and Fe(BTC) (Basolite F300) were provided by Sigma Aldrich.

### Characterization

The crystallinity of samples under study was determined by X-ray powder diffraction on a Bruker AXS D8 Advance diffractometer with a Vantec-1 detector in the Bragg-Brentano geometry using CuKα radiation. To limit the effect of preferential orientation of individual crystals a gentle grinding of the samples was performed before measurements.

Adsorption isotherms of nitrogen at −196°C were determined using an ASAP 2020 (Micromeritics) static volumetric apparatus. In order to attain sufficient accuracy in the accumulation of the adsorption data, the ASAP 2020 was equipped with pressure transducers covering the 133 Pa, 1.33 kPa and 133 kPa ranges. Before adsorption experiments the samples were outgassed under turbomolecular pump vacuum at temperature of 150°C for MOFs and 250°C for zeolites. This temperature was maintained for 8 h.

The concentrations of Brønsted and Lewis acid sites in zeolites were determined by pyridine adsorption at 150°C followed by FTIR spectroscopy (Nicolet 6700) using self-supporting wafer technique. Generally, a thin sample wafer of zeolite was activated prior to the experiment in a high vacuum (10^−4^ Torr) at 450°C overnight. Adsorption of pyridine proceeded at room temperature for 30 min at a partial pressure of 5 Torr and was followed by 20 min evacuation at the temperature 150°C. For the quantitative determination of concentration of relevant acid sites the molar adsorption coefficients (Emeis, 1993) for pyridine adsorbed on Brønsted [ν(C = N) − B at 1540 cm^−1^, ε(Br) = 1.67 cm/μmol] and Lewis acid sites [ν(C = N) − L at 1470 cm^−1^, ε(L) = 2.22 cm/μmol] were used. Determination of Lewis acid sites in CuBTC is discussed in detail elsewhere (Pérez-Mayoral et al., 2012).

### Catalysis

The condensation reactions were performed in a liquid phase under atmospheric pressure in a multi-experiment work station StarFish (Radley's Discovery Technologies UK). Prior to use, 200 mg of the catalyst was activated at 150°C (for MOFs) or 450°C (for zeolites) for 90 min with a temperature rate 10°C/min in a stream of air.

#### Pechmann condensation

Typically, 8.5 mmol of 1-naphthol, 0.5 g of n-dodecane (internal standard), 10 ml of nitrobenzene and 200 mg of catalyst were added to the 3-necked vessel, equipped with condenser and thermometer, stirred, and heated. 100 mmol of ethyl acetoacetate was added into the reaction vessel through a syringe when the temperature of 130°C was reached.

#### Knoevenagel condensation

Typically, 6.0 mmol of aldehyde, 0.4 g of mesitylene (internal standard), 10 ml of p-xylene and 200 mg of catalyst were added to the 3-necked vessel, equipped with condenser and thermometer, stirred, and heated. 9.0 mmol of ethyl acetoacetate was added into the reaction vessel through a syringe when the temperature of 130°C was reached.

#### Prins condensation

Typically, 8.0 mmol of PF, 0.4 g of mesitylene (internal standard), 10 ml of acetonitrile and 200 mg of catalyst were added to the 3-necked vessel, equipped with a condenser and a thermometer, stirred, and heated. 4.0 mmol of β-pinene was added into the reaction vessel through a syringe when the temperature of 80°C was reached.

Aliquots of the reaction mixture were sampled at the interval time of 0, 20, 60, 120, 180, 240, 300, 360 min in order to determine the equilibrium of the reaction. Zero point of conversion corresponds to the concentration of phenol, aldehyde, or β-pinene in starting solution in the presence of catalyst (to neglect the contribution of adsorption).

To evaluate a potential influence of leaching of active species from the heterogeneous catalysts, a part of the reaction mixture was filtered at the reaction temperature and the obtained liquid phase was further investigated in condensation reaction under the same reaction conditions.

#### Reaction product analysis

The reaction products were analyzed by gas chromatography (GC) using an Agilent 6850 with FID detector equipped with a non-polar HP1 column (diameter 0.25 mm, thickness 0.2 μm and length 30 m). Reaction products were indentified using GC-MS analysis (ThermoFinnigan, FOCUS DSQ II Single Quadrupole GC/MS).

## Results and discussion

### Characteristics of the catalysts

**Figure A1:**
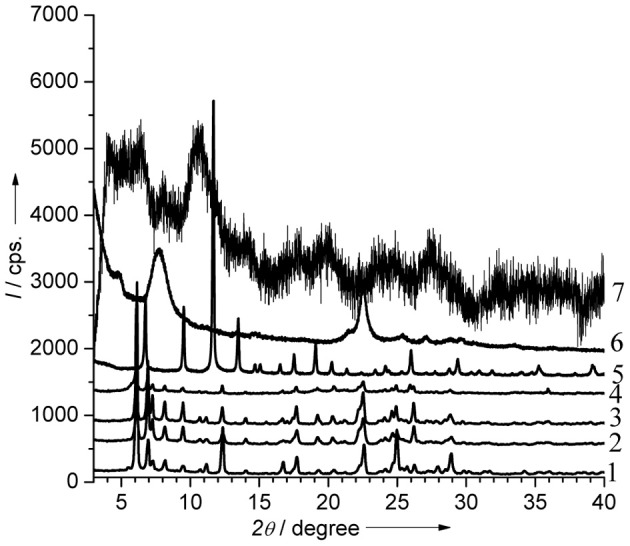
**X-ray powder diffraction patterns of the catalysts**. 1—(B)UTL, 2—(Al)UTL, 3—(Ga)UTL, 4—(Fe)UTL, 5—Cu_3_(BTC)_2_, 6—(Al)beta, 7—Fe(BTC).

The X-ray diffraction patterns of all the catalysts match well with those reported in the literature (Figure A1) (Chui et al., 1999; Shvets et al., 2008; Dhakshinamoorthy et al., 2012).

While (Al)beta, (B), (Al), (Ga), (Fe)UTL and Cu_3_(BTC)_2_ were found to be highly crystalline, Fe(BTC) represents less ordered material.

**Figure 1:**
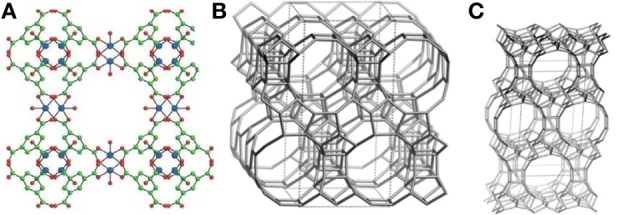
**Frameworks of Cu_3_(BTC)_2_ (A), beta (B), UTL (C)**.

The known frameworks of the catalysts under investigation are depicted on Figure 1. In Cu_3_(BTC)_2_ framework, the Cu^2^-clusters are coordinated via carboxylate groups of benzene-1,3,5-tricarboxylate to form a paddlewheel unit in a three-dimensional porous cubic network (Figure 1A). Zeolite beta consists of an intergrowth of two distinct structures termed polymorphs A (Figure 1B) and B. The polymorphs grow as two-dimensional sheets and the sheets randomly alternate. Both polymorphs have a three dimensional network of 12-ring pores (0.64 × 0.76 and 0.56 × 0.56 nm). The intergrowth of the polymorphs does not significantly affect the pores in two of the dimensions, but in the direction of the faulting, the pore becomes tortuous, but not blocked. Zeolite UTL (Figure 1C) belongs to the extra-large pore zeolites having 2D pore system of intersecting 14- (0.71 × 0.95 nm) and 12-ring channels (0.85 × 0.55 nm).

**Figure 2:**
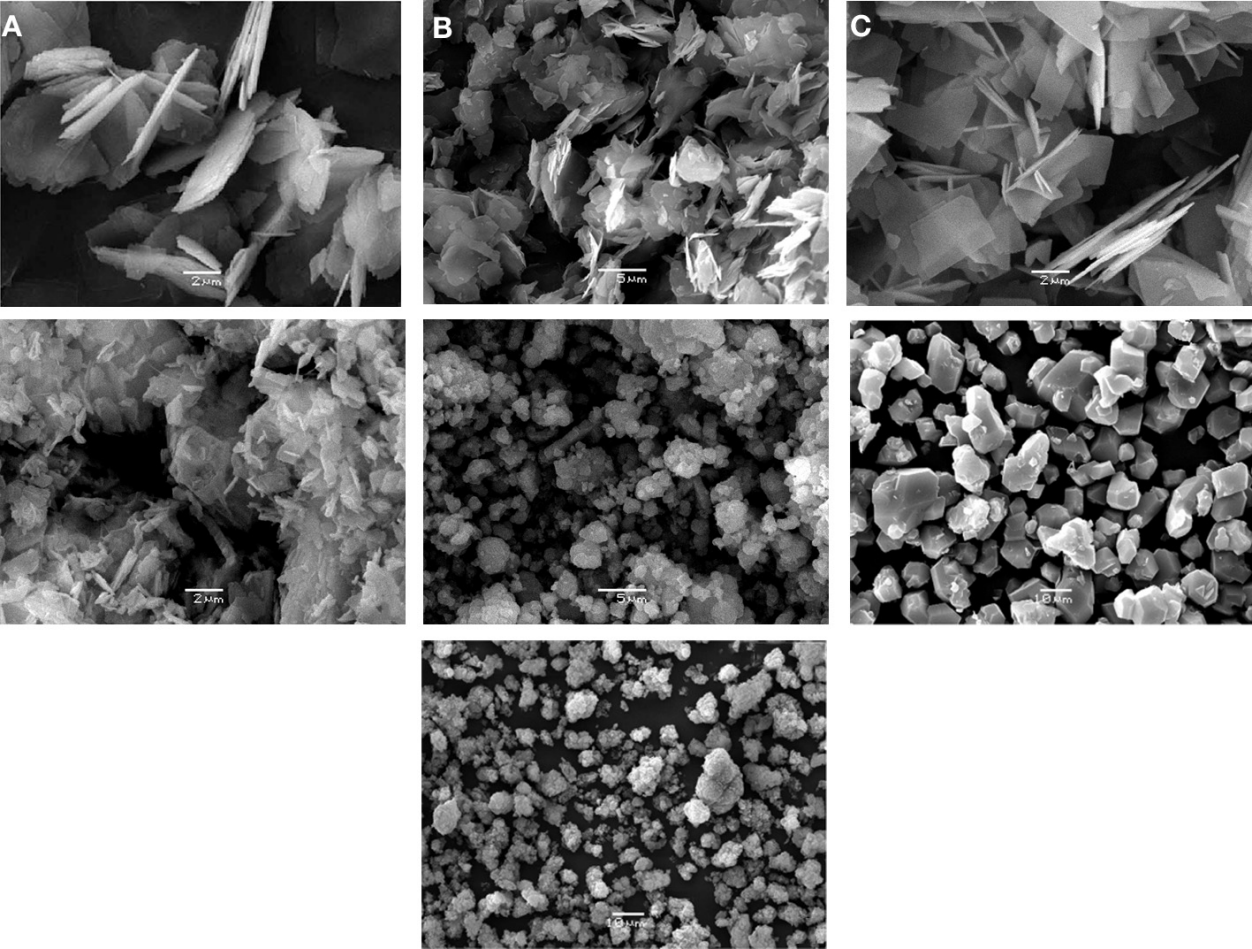
**SEM images of the catalysts**. **(A)** (B)UTL, **(B)** (Al)UTL, **(C)** (Ga)UTL, **(D)** (Fe)UTL, **(E)** (Al)beta, **(F)** Cu_3_(BTC)_2_, **(G)** Fe(BTC).

Crystals of UTL zeolites possess rectangular shape (Figure 2) and have the close size, except (Al)UTL (Table 1). Crystals of zeolite (Al)beta are characterized by size about 0.5 μm. The crystals of Cu_3_(BTC)_2_ are rectangular prisms with the length of the edges of about 7 μm, while the size of the crystals of Fe(BTC) is about 3 μm.

**Table 1:** **Textural properties of the catalysts**.

**Catalyst**	**Crystal size^[a]^, μm**	***S*_BET^[b]^_, m^2^/g**	***D*_micro^[b]^_, nm**	***V*_micro^[b]^_, cm^3^/g**
Cu_3_(BTC)_2_	7	1500	0.90	0.64
Fe(BTC)	3	1060	0.86	0.33
(B)UTL	6.0 × 4.0 × 0.2	570	1.00	0.21
(Ga)UTL	7.0 × 5.0 × 0.2	450	1.00	0.17
(Fe)UTL	6.0 × 4.0 × 0.5	550	1.00	0.21
(Al)UTL	4.0 × 0.5 × 0.1	500	1.00	0.19
(Al)beta	0.5	670	0.66	0.2

[a] *According to SEM images*.

[b] *According to adsorption/desorption isotherms of N_2_*.

**Figure 3:**
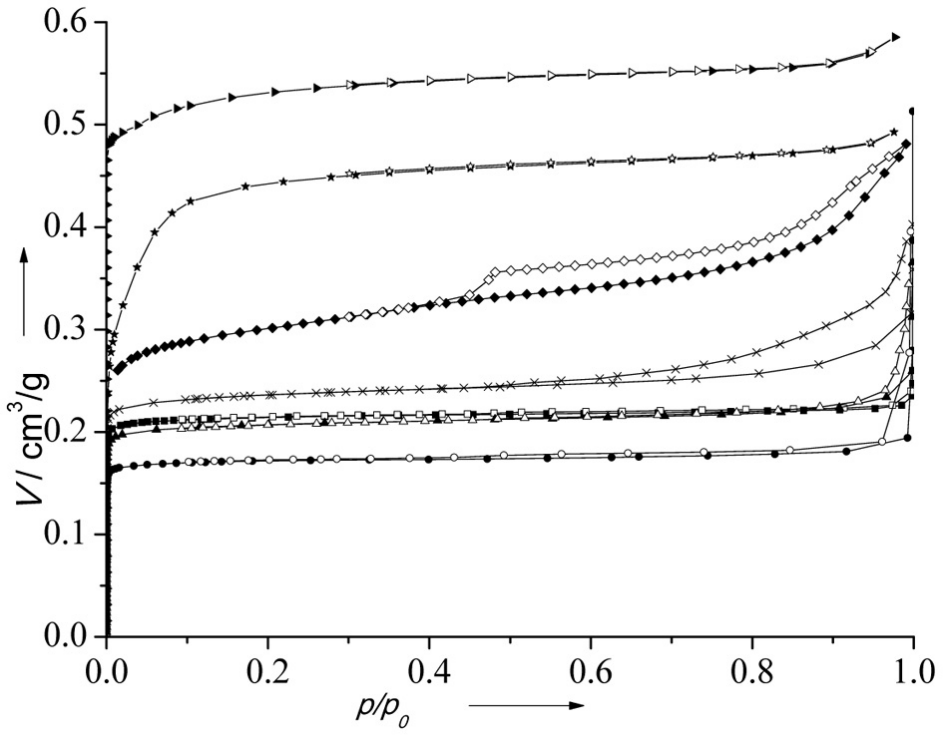
**N_2_ adsorption isotherms of the catalysts**. (B)UTL (–■–), (Ga)UTL (–•–), (Fe)UTL (–▲–), (Al)UTL (–X–), (Al)beta (–♦–), Fe(BTC) (–★–), Cu_3_(BTC)_2_ (–▶–). Full points represent adsorption, open points—desorption.

Nitrogen adsorption isotherms of the catalysts are depicted in Figure 3. All catalysts exhibit type I isotherm being characteristic for microporous solids. The presence of a hysteresis loop at *p* > 0.8 on the isotherm of (Al)UTL is probably connected with an interparticle adsorption. Textural properties of all catalysts are summarized in Table 1.

Acidic properties of the catalysts (i.e., type and concentrations of acid sites) were analyzed using adsorption of pyridine followed by FTIR. For zeolites, absorption bands around 1546 cm^−1^ (interaction of pyridine with Brønsted acid sites) and 1453–1455 cm^−1^ (interaction of pyridine with Lewis acid sites) were chosen as characteristic. To estimate the acidity of the Cu_3_(BTC)_2_ band at 1069 cm^−1^ assigned to C–C out-of-plane vibrations of coordinatively bonded pyridine was chosen (Pérez-Mayoral et al., 2012). Characteristic bands in region 1400–1600 cm^−1^ were not used for the determination of the amount of acid sites in MOFs because of an overlap with the bands corresponding to MOF's framework.

Table 2 lists the concentrations of Brønsted and Lewis acid sites for different temperatures of pyridine desorption.

**Table 2:** **Acid properties of the catalysts**.

**Catalyst**	***T*^III^^[a]^ (mol. %)**	***T*^IV^/*T*^III^**	**IR**		
			***T*_des_. (°C)**	**Brønsted, (μmol/g)**	**Lewis, (μmol/g)**
(B)-UTL	0.5	199	150	15	60
			250	15	41
			350	0	0
			450	0	0
(Ga)UTL	1.4	70	150	23	64
			250	21	62
			350	14	57
			450	6	61
(Fe)UTL	5.9	16	150	36	97
			250	19	35
			350	12	31
			450	7	22
(Al)UTL	2.1	47	150	69	61
			250	49	48
			350	31	44
			450	20	41
(Al)beta	7.4	12.5	150	210	320
			250	180	240
			350	120	220
			450	50	180
Cu_3_(BTC)_2_	–	–	–	–	2300

[a] *According to the chemical analysis*.

It can be seen, that the concentration of acid sites increases in the following sequence: (B) < (Fe) < (Ga) < (Al)UTL << (Al)beta. The concentration of coordinatively bonded pyridine was determined to be equal to 2.30 mmol g^−1^ for Cu_3_(BTC)_2_ activated at 200°C (Pérez-Mayoral et al., 2012).

### Knoevenagel condensation

The catalytic performance of MOFs and isomorphously substituted extra-large pore zeolites UTL was studied in Knoevenagel condensation of cyclohexane carbaldehyde (ChCA) and benzaldehyde (BA) with ethylacetoacetate (EAA) and compared with large-pore alumosilicate zeolite (Al)beta.

**Scheme 4:**
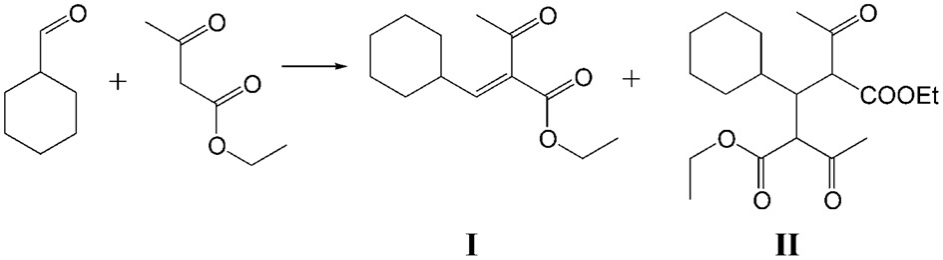
**Condensation of ChCA with EAA**.

**Scheme 5:**
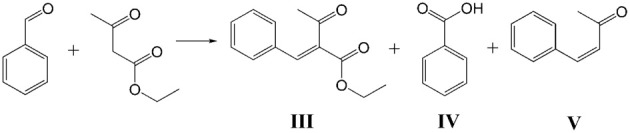
**Condensation of BA with EAA**.

Ethyl 2-(cyclohexylmethylene)-3-oxobutanoate (product **I**) and diethyl 2,4-diacetyl-3-cyclohexylpentanedioate (product **II**) were detected as the main products of Knoevenagel condensation of ChCA with EAA by GC-MS (Scheme 4) while ethyl 2-benzylidene-3-oxobutanoate (product **III**), benzoic acid (product **IV**) and 4-phenylbut-3-en-2-one (product **V**) were found in the case of benzaldehyde (BA) condensation (Scheme 5).

**Figure 4:**
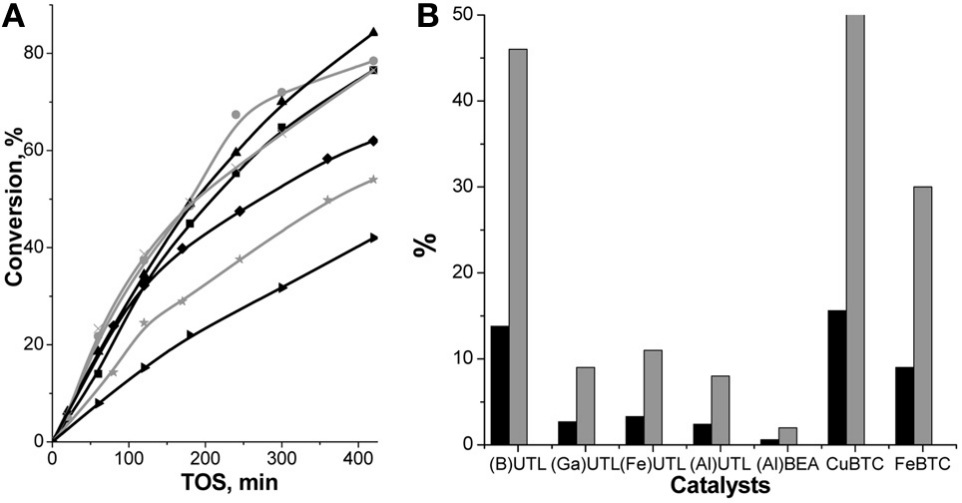
**TOS dependence of ChCA conversion in condensation reaction with EAA over (B)UTL (–■–), (Ga)UTL** 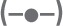
**, (Fe)UTL (–▲–), (Al)UTL** 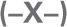
**, (Al)beta (–♦–), Fe(BTC)** 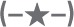
**, Cu_3_(BTC)_2_ (–▶–) (A); the yield (■) and selectivity** 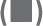 **(at 30% of ChCA conversion) to the product I (B)**.

**Figure 5:**
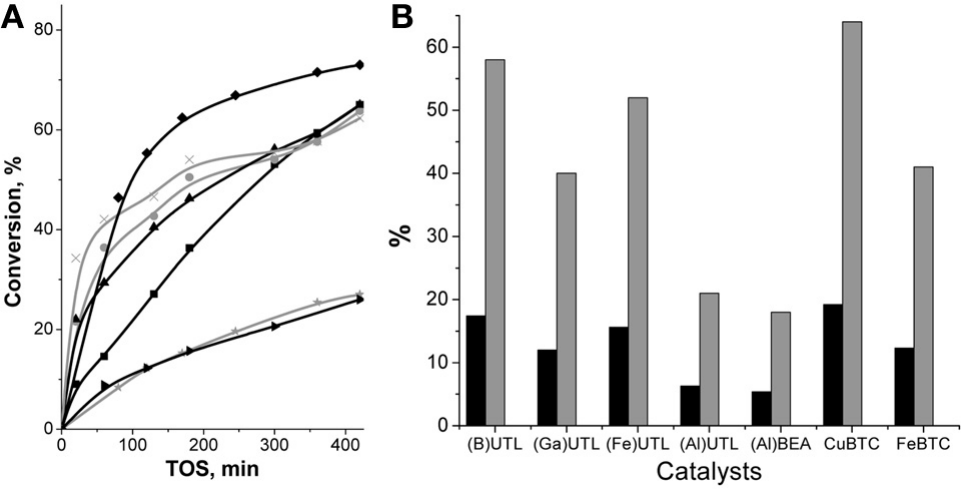
**TOS dependence of BA conversion in condensation reaction with EAA over (B)UTL (–■–), (Ga)UTL** 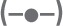
**, (Fe)UTL (–▲–), (Al)UTL** 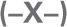
**, (Al)beta (–♦–), Fe(BTC)** 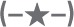
**, Cu_3_(BTC)_2_ (–▶–) (A); the yield (■) and selectivity** 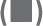 **(at 15% of BA conversion) to the product III in condensation of BA with EAA (B)**.

The conversion of aldehydes increased over UTL zeolites in the following sequence: (B) < (Fe) < (Ga) < (Al)UTL (Figures 4A, 5A) matching well with the increasing concentration of accessible acid sites and particularly their acid strength. At the same time, while Fe-, Ga- and Al-substituted UTL zeolites appeared to be low-selective catalyst (Figure 4B) in condensation of ChCA with EAA (11.9 and 8% selectivity to product I, respectively), the selectivity 46% of ether I was achieved when using B-containing UTL zeolite as the catalyst. This is most probably related to the acceleration of the rate of secondary reactions, involving the primary product I, with increasing the concentration of acid sites in the range (B) < (Fe) ≈ (Ga) < (Al)UTL.

The initial conversions of ChCA in condensation reaction with EAA over (Al)beta and (Al)UTL were quite similar (23% at 60 min TOS, Figure 4A). With increasing TOS the slope of the curve “conversion–TOS” became flatter for (Al)beta indicating a lowering of the reaction rate. It may be caused by diffusion restrictions for substrates/products in the porous system of zeolite beta in comparison with extra-large pore zeolite UTL despite the crystal size of (Al)beta is much more lower in comparison with (Al)UTL (0.5 × 0.5 × 0.5 vs. 4.0 × 0.5 × 0.1 μm, Table 1). Moreover, the selectivity of (Al)beta possessing the highest concentration of strong acid sites, reached expectedly the lowest value (2%) among the investigated catalysts (Figure 4B).

Surprisingly, the lowest conversion of ChCA was found for Cu_3_(BTC)_2_ (Figure 4A), possessing comparably high concentration of mild Lewis acid sites (Table 2). This may be due to the relative weakness of the respective tetracoordinated Cu^2+^ sites within the MOF for the activation of ChCA. However, Cu_3_(BTC)_2_ demonstrated higher selectivity to the product **I** in comparison with UTL zeolites (52%, Figure 4B), which may be also caused by low strength of acid sites within Cu_3_(BTC)_2_.

When compared with ChCA, BA appeared to be easier activated substrate—the conversions of BA over all investigated catalysts were comparable or exceeded the respective values for ChCA (Figures 4A, 5A).

At the same time the increasing selectivity of the catalysts in the case of BA condensation with EAA may be caused by the electron-withdrawing effect of phenyl-group. It results in decreasing reactivity of the primary product **III** (Scheme 5) in the secondary reactions of Michael addition in comparison with the product **I** being transformed to product **II** (Scheme 4) over all investigated catalyst. Even though, Al-substituted zeolites beta and UTL were characterized by the lowest selectivity (20%, Figure 5B) due to a rapid hydrolysis of the target ether **III** on strong Brønsted acid sites followed by dicarboxylation with the formation of side-product **IV** (Scheme 5). At the same time, the product of BA oxidation—benzoic acid was found as the side product when using (B)UTL, Cu_3_(BTC)_2_, Fe(BTC), which appeared to be the most selective among the investigated ones.

**Figure 6:**
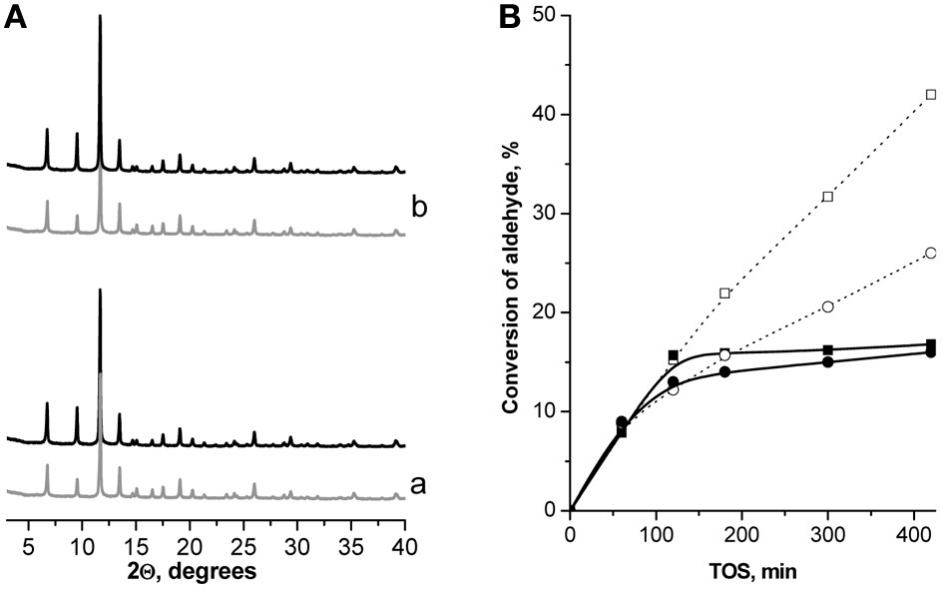
**XRD patterns of Cu_3_(BTC)_2_ before (−) and after** 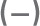 **24 h TOS in Knoevenagel condensation of ChCA (a) and BA (b) with EAA at 130°C (A); leaching test: TOS dependence of the conversion of ChCA (–■–) and BA (–•–) in Knoevenagel condensation with EAA (full line—leaching test, dotted line—standard experiment, *T* = 130°C) (B)**.

The yields of the target products in Knoevenagel condensation of both ChCA and BA with EAA over the catalysts under investigation increase in the following sequence: (Al)beta < (Al)UTL < (Ga)UTL < (Fe)UTL < Fe(BTC) < (B)UTL < Cu_3_(BTC)_2_, which is caused by the enhancement of the catalyst's selectivity with the decreasing of the strength of active sites. The most active Cu_3_(BTC)_2_ catalyst was proved to be structurally stable upon liquid phase Knoevenagel condensation with EAA at 130°C when using p-xylene as the solvent (Figure 6A). In addition, a negligible increase in the conversion of initial aldehydes after removing of Cu_3_(BTC)_2_ from the “hot” reaction mixture at 120 min TOS confirms the absence of homogeneous reaction contribution, which might be caused by the leaching of active sites to the liquid phase (Figure 6B).

Despite Knoevenagel reaction can be catalyzed by both solid acids (activation of carbonyl component) and bases (activation of methylene component) (Tietze and Beifuss, 1991), it was mainly tested over basic catalysts. In ref. (Lau et al., 2008), Knoevenagel condensation of BA and EAA was performed in a microreactor using Cs-exchanged NaA membrane and NaX crystals. It was shown that the using catalyst film instead of powder results in suppression of undesirable side-reactions (increasing the selectivity from 55 to 78%) due to the rapid elaboration of Knoevenagel adduct from the porous system of the catalyst. Analogously, poor selectivity of (Al)beta in comparison with (Al)UTL may be caused by diffusional restrictions for the removal of the product from the porous system of the first catalyst. The influence of the strength of basic sites on the activity of Li-, Na-, K-, Rb-, Cs-impregnated simple oxide matrices (SiO_2_, Al_2_O_3_, Nb_2_O_5_) was established (Calvino-Casilda et al., 2009). The best performance of K/Al_2_O_3_ in Knoevenagel condensation of BA and EAA (90% of BA conversion and 89.8% of selectivity at 140°C in 300 min of TOS) was connected with the mild strength of basic sites, found for this system (Calvino-Casilda et al., 2009). Analogously, in the range of Nb-MCM-41 catalysts, impregnated by alkali metals, Rb/Nb-MCM-41 demonstrated the best performance (80% of benzaldehyde conversion in 4 h of TOS at 140°C) (Calvino-Casilda et al., 2010).

### Pechmann condensation

**Figure A2:**
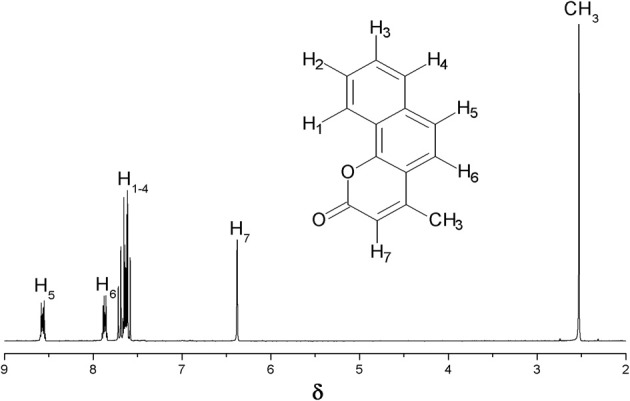
**^1^HNMR spectrum for 4-methyl-2H-benzo[h]-chromen- 2-one obtained over Fe(BTC) and Cu_3_(BTC)_2_**.

Pechmann condensation of 1-naphthol proceeds according to the Scheme 2 with the formation of 4-methyl-2H-benzo[h]chromen-2-one (Figure A2) as the target product and some unidentified polycondensation products.

**Figure 7:**
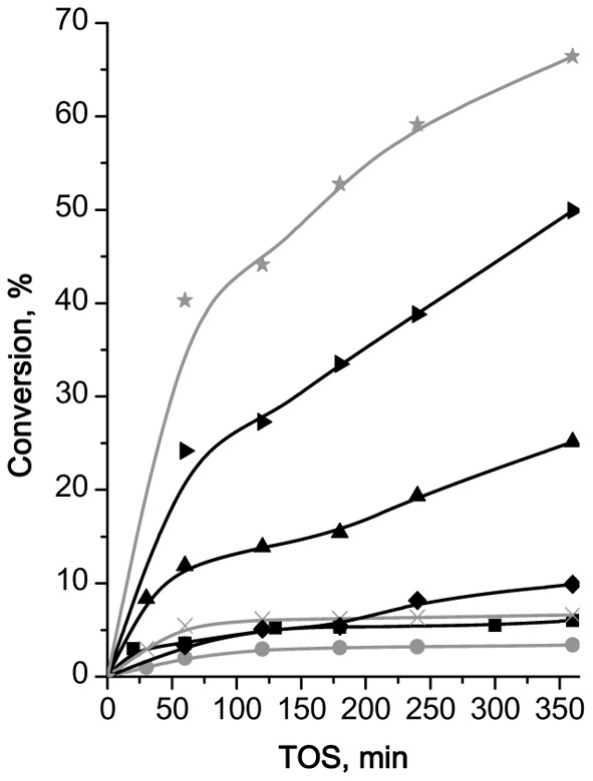
**TOS dependence of conversion of 1-naphthol in Pechmann condensation reaction with EAA over (B)UTL (–■–), (Ga)UTL** 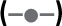
**, (Fe)UTL (–▲–), (Al)UTL** 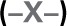
**, (Al)beta (–♦–), Fe(BTC)** 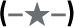
**, Cu_3_(BTC)_2_ (–▶–)**.

Superiority of MOFs over large-pore zeolite (Al)beta and extra-large pore zeolite UTL in Pechmann condensation of 1-naphthol was observed. While the conversion of the initial substrate at 120 min TOS did not exceed 15% for zeolites, it totaled 33 and 44% for Cu_3_(BTC)_2_ and Fe(BTC), respectively (Figure 7). The selectivity to the target product was about 98% for both MOFs. A lower initial reaction rate over Cu_3_(BTC)_2_ in comparison with Fe(BTC) may be caused by a lower accessibility of the active sites within Cu_3_(BTC)_2_ catalyst, possessing smaller pore windows in comparison to Fe(BTC) (Table 1).

Higher conversion of 1-naphthol in Pechmann condensation over MOFs in comparison with zeolites may be connected with significantly higher concentration of acid sites of sufficient strength to activate the substrate. In addition, the facilitation of transport of both reagents and product within pore system of MOFs and extra-large pore UTL zeolites, most likely, prevents side-transformations resulting in 100% selectivity to the target product of 1-naphthol condensation with EAA. Only 80% selectivity was observed when (Al)beta was used.

**Figure A3:**
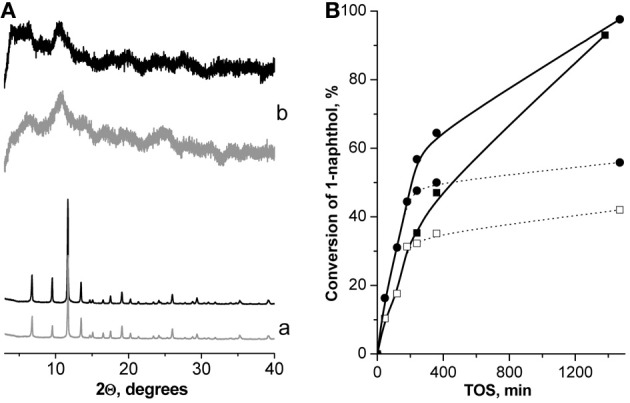
**XRD patterns of Cu_3_(BTC)_2_ (a) and Fe(BTC) (b) before (−) and after (−) 24 h TOS in Pechmann condensation of 1-naphthol at 130°C (A); leaching test: TOS dependence of the conversion of 1-naphthol in Pechmann condensation of 1-naphthol (full line—standard experiment, dotted line—leaching test, *T* = 130°C) for –■– Cu_3_(BTC)_2_, –•– Fe(BTC) (B)**.

XRD patterns of Cu_3_(BTC)_2_, elaborated from the reaction mixture after 24 h TOS, provide an evidence of the preservation of the framework of the MOF during the Pechmann condensation of 1-naphthol. At the same time no significant differences in the position of diffraction lines for the fresh and utilized Fe(BTC) were observed (Figure A3A).

Leaching test of the investigated MOFs removing them from the reaction mixture by “hot” centrifugation was not accompanied by the further increase in the conversion of 1-naphthol (Figure A3B), which strongly denies the leaching of the active sites for Cu_3_(BTC)_2_ and Fe(BTC).

It should be noted that catalytic activity of and FeBTC is comparable with periodic mesoporous silica chloride (the yield of target product 75% at 130°C and 180 min of TOS),(Karimi and Behzadnia, 2011) and SBA-15-Ph-Pr-SO_3_H (the yield of target product 65% at 130°C and 240 min of TOS) (Kalita et al., 2010), which were shown to be one of the most promising catalysts for Pechmann condensation of 1-naphthol.

### Prins reaction

**Scheme 6:**
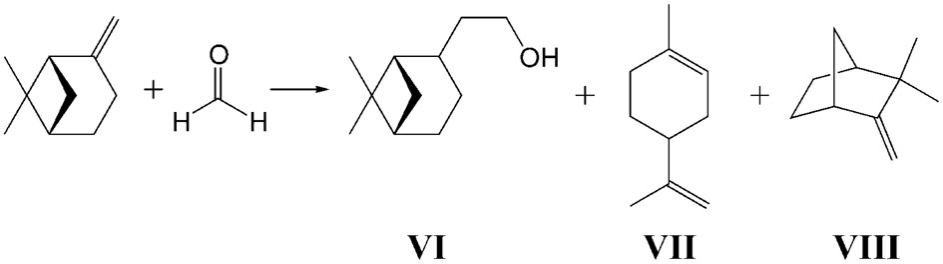
**Prins condensation of β-pinene with PF**.

In the Prins reaction of β-pinene with formaldehyde besides the formation of target product nopol (**VI**) we observed also the formation of the side products of β-pinene isomerization—limonene (**VII**) or camphene (**VIII**) (Scheme 6).

**Figure 8:**
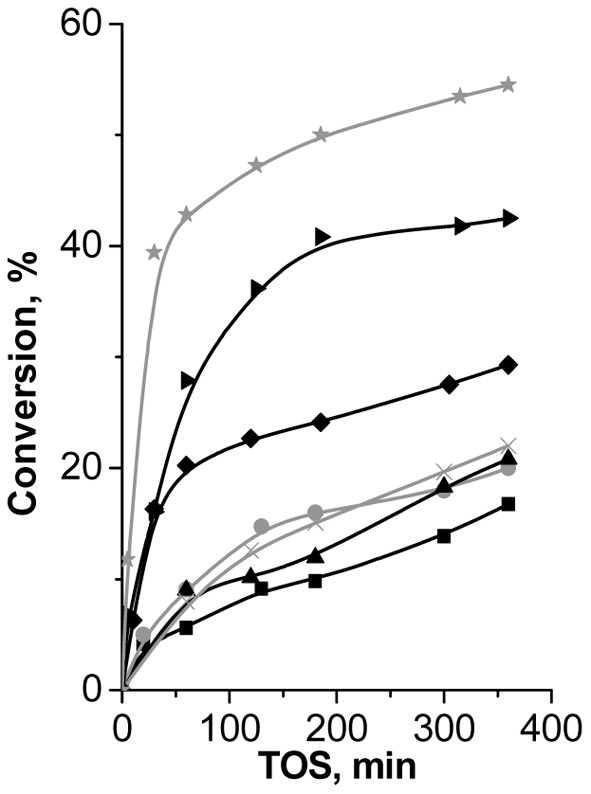
**TOS dependence of conversion of β-pinene in Prins reaction with PF over (B)UTL (–■–), (Ga)UTL** 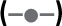
**, (Fe)UTL (–▲–), (Al)UTL** 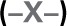
**, (Al)beta (–♦–), Fe(BTC)** 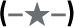
**, Cu_3_(BTC)_2_ (–▶–)**.

Fe(BTC) showed the highest performance (47% of β-pinene conversion at 120 min of TOS, Figure 8) in Prins reaction of β-pinene with formaldehyde among the investigated catalysts and providing 100% selectivity to the target product—nopol.

Both isomorphously substituted UTL and (Al)beta zeolites appeared to be less efficient catalysts in reaction of β-pinene with formaldehyde in comparison with Fe(BTC). It is obviously caused by the much lower concentration of active sites in zeolites (Table 2). The conversion of β-pinene increases for extra-large-pore UTL zeolites in the sequence: (B) < (Fe) < (Ga) ≈ (Al)UTL and does not exceed 15% in 120 min of TOS (Figure 8). The conversion of β-pinene over large-pore zeolite (Al)beta was higher than over UTL-based catalysts, which might be due to the higher concentration of active sites accessible for β-pinene (Table 2). In contrast to Fe(BTC), we observed the formation of side product **VI** over zeolite beta (40% selectivity to nopol). This result shows that the presence of relatively strong Brønsted acid sites leads to the reduction of the catalyst performance in reaction of β-pinene with formaldehyde.

Surprisingly, only isomerization of initial substrate with the formation of camphene took place over Cu_3_(BTC)_2_, which similarly to Fe(BTC) is characterized by the mild exclusively Lewis acidity and close size of micropores. The reasons of camphene formation are not entirely clear on the moment.

**Figure A4:**
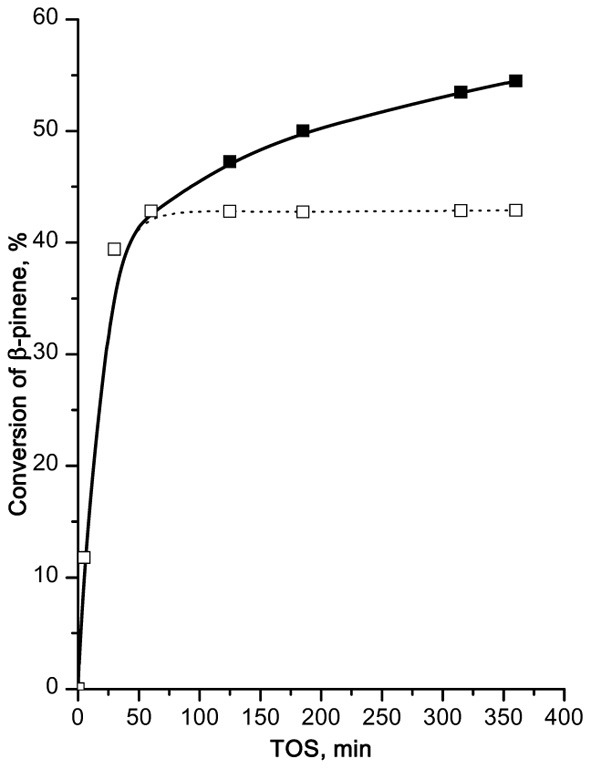
**Leaching test: TOS dependence of the conversion of β-pinene in Prins condensation with PF (full line—standard experiment, dotted line—leaching test, *T* = 80°C) for Fe(BTC)**.

To check the possibility of leaching of Fe^3+^ ions from the framework to the solution, the most active Fe(BTC) catalyst was surveyed for leaching, which evidences a negligible increase in the conversion after removal by centrifugation of the solid catalyst from reaction mixture at 120 min of TOS, indicating that no leaching of the active species takes place (Figure A4).

Thus, Fe(BTC), containing the largest amount of acid sites of appropriate strength to activate initial β-pinene, overtops large-pore zeolite (Al)beta and B-, Al-, Ga-, Fe-substituted extra-large pore zeolites UTL in Prins reaction of β-pinene with PF.

When compared our results with the previously obtained, montmorillonite impregnated with ZnCl_2_ prepared by wet impregnation technique was shown to be active in Prins reaction of β-pinene with PF (75% of β-pinene conversion and 97% selectivity after 24 h TOS). However, leaching of Zn^2+^ took place, contributing into the overall conversion value (Yadav and Jasra, 2006). In (Selvaraj and Choe, 2010) Sn-doped SBA-15 was shown to be highly active catalyst for nopol synthesis via Prins reaction of β-pinene and PF. The activity of the catalyst increased with the increasing of the concentration of active tetracoordinated Sn^4+^ species. The best results (β-pinene conversion of 99.3% and a nopol selectivity of 95.4% at 360 min of TOS) were achieved over Sn-SBA-15 (Si/Sn = 5), which was characterized by the highest amount of Lewis acid centers.

## Conclusions

Catalytic performance of extra-large pore germanosilicate UTL zeolites prepared by incorporation of different heteroelements (e.g., B, Al, Ga, Fe) into the framework in Knoevenagel, Pechmann, Prins reactions was investigated and compared with that of large-pore aluminosilicate zeolite beta and Cu-, Fe-containing metal-organic-frameworks.

The performance of UTL zeolites in Knoevenagel condensation is mainly determinated by the strength of acid sites. While the conversion of initial ChCA and BA matches with the total concentration of acid sites and increases in the following sequence (B) < (Fe) < (Ga) < (Al)UTL, the selectivity of the catalysts depends on the strength of active centers and has the highest value for B-UTL, containing the weakest acid centers.

The yields of the target products in Knoevenagel condensation of both ChCA and BA with EAA over the catalysts under investigation increase in the following sequence: (Al)beta < (Al)UTL < (Ga)UTL < (Fe)UTL < Fe(BTC) < (B)UTL < Cu_3_(BTC)_2_, which is caused by the enhancement of the catalyst selectivity with the decreasing of the strength of active sites.

Fe(BTC) surpasses not only large-pore zeolite (Al)beta, but also isomorphously substituted extra-large pore zeolites UTL in Pechmann condensation of 1-naphthol with EAA and Prins reaction of β-pinene with PF, which seems to be caused by the much more higher concentration of accessible active sites within MOF and their appropriate strength.

### Conflict of interest statement

The authors declare that the research was conducted in the absence of any commercial or financial relationships that could be construed as a potential conflict of interest.
